# Quality of life and phonatory and morphological outcomes in cognitively unimpaired adolescents with Pierre Robin sequence: a cross-sectional study of 72 patients

**DOI:** 10.1186/s13023-021-02072-0

**Published:** 2021-10-20

**Authors:** Béatrice Thouvenin, Véronique Soupre, Marie-Anne Caillaud, Charlotte Henry-Mestelan, Christel Chalouhi, Bachar Houssamo, Cécile Chapuis, Katia Lind, Aurélie Royer, Nancy Vegas, Jeanne Amiel, Gérard Couly, Arnaud Picard, Laurence Vaivre-Douret, Véronique Abadie

**Affiliations:** 1 General Paediatrics Unit, Necker University Hospital, APHP, 149 rue de Sèvres, 75015 Paris, France; 2 Referral Centre for Rare Diseases “Syndrome de Pierre Robin et troubles de succion-déglutition congénitaux», Necker University Hospital, APHP, Paris, France; 3 Paediatric Maxillofacial and Plastic Surgery Unit, Necker University Hospital, APHP, Paris, France; 4 IQVIA, Paris, France; 5 Imagine Institute, Paris, France; 6 Genetics Department, Necker University Hospital, APHP, Paris, France; 7 Paris University, Paris, France; 8 INSERM Unit 1178, CESP, Paris, France

**Keywords:** Pierre Robin sequence, Generic quality of life, Oral quality of life, Vocal quality of life, Outcome

## Abstract

**Background:**

Pierre Robin sequence (PRS) is a heterogeneous condition involving retro(micro)gnathia, glossoptosis and upper airway obstruction, very often with posterior cleft palate. Patients with PRS, either isolated or associated with Stickler syndrome have good intellectual prognosis. Nevertheless, the quality of life in adolescence and the phonatory and morphological outcomes are rarely analysed. We assessed the phonatory and morphological outcomes of 72 cognitively unimpaired adolescents with PRS, studied their oral (COHIP-SF19), vocal (VHI-9i) and generic quality of life (QoL; KIDSCREEN-52), and searched for determinants of these outcomes.

**Results:**

Two-thirds of our adolescents retained low or moderate phonation difficulties, but risk factors were not identified. For 14%, morphological results were considered disharmonious, with no link to neonatal retrognathia severity. Only one vs two-stage surgery seemed to affect final aesthetic results. The oral QoL of these adolescents was comparable to that of control patients and was significantly better than that of children with other craniofacial malformations (COHIP-SF19 = 17.5, 15.4 and 25.7, respectively). The oral QoL of the adolescents with non-isolated PRS was significantly worse (COHIP-SF19 = 24.2) than that of control patients and close to that of children with other craniofacial malformations. The vocal QoL of the adolescents (mean [SD] VHI-9i = 7.5 [5.4]) was better than that of patients with other voice pathologies and better when phonation was good. The generic QoL of the adolescents was satisfactory but slightly lower than that of controls, especially in dimensions concerning physical well-being, relationships and autonomy. QoL results were lower for adolescents with non-isolated than isolated PRS. Only non-isolated PRS and low oral QoL affected generic QoL.

**Conclusion:**

Morphological or phonatory impairments remain non-rare in adolescents with PRS but do not seem to be directly responsible for altered QoL. These adolescents, especially those with non-isolated PRS, show self-confidence and social-relation fragility. We must focus on long-term functional and psychological results for PRS patients and improve therapy protocols and follow-up, notably those affecting the oral aspects of the disease.

## Introduction

Pierre Robin sequence (PRS) is a rare and complex facial malformation that occurs in approximately 1 in 10,000 births. It associates retro(or micro)gnathia, glossoptosis, airway obstruction and frequently posterior U-shaped cleft palate (CP) [1]. The pathophysiological origin of the embryonic sequence of events leading to the disorder seems heterogeneous [2, 3]. A family history of PRS is present in 10% to 15% of cases, possibly involving mutations upstream of the SOX9 gene, which participates in embryonic mandible development among other roles [4, 5]. Neonates with PRS exhibit breathing and feeding issues, including upper airway obstruction due to the posterior position of the tongue and to glossopharyngeal/laryngeal hypotonia; sucking and swallowing difficulties; and gastroesophageal reflux [6, 7]. Because of these functional disorders, the first year for an infant with PRS is marked by many burdensome medical necessities, such as prolonged hospitalization, mother/child separation, nasogastric or gastrostomy tube feeding and management of upper airway obstruction. In the Paris hospital protocol, this latter management comprises prone positioning, continuous positive airway pressure, nasopharyngeal tube insertion, or tracheostomy according to case severity [8, 9]. Patients usually improve progressively during the first 2 years of life. In PRS with CP, one or two-stage primary surgery, according to the surgical team and the CP width, is performed between age 6 and 18 months.

Nearly half of children with PRS also have associated malformations resulting in syndromic PRS often affecting their cognitive development. In contrast, those with isolated PRS or PRS associated with Stickler syndrome have good cognitive prognosis. However, in a previous prospective longitudinal study spanning 12 years, we showed that about half of such children had good cognitive development but retained phonatory disorders, especially hypernasality [10].

A number of teams have looked at the negative impact of cleft lips/palates on quality of life (QoL), particularly in terms of self-esteem [11–14]. In contrast, only two studies have been published on QoL in PRS patients [15, 16]. The authors of the first study, concerning younger children (age 4–11 years), reported that self-esteem was comparable in the PRS and control group but underlined that this dimension should be evaluated in adolescents. Results of the more recent second study demonstrated a satisfactory generic QoL in 17 adolescents with PRS. Nonetheless, both families and health providers report self-esteem issues, inhibitions, and social integration difficulties in some teenagers affected by PRS, even those with normal cognitive and scholastic capacities. The hypernasality and face morphology issues they may still carry could contribute to these issues because the voice and face are major elements of social communication.

The primary objective of our study was to analyse generic and specific (vocal and oral) QoL in cognitively unimpaired adolescents with PRS, and identify determinants of any differences between QoL in this series and the general population. Those determinants were defined as current, particularly phonatory or facial morphology sequelae still present; or early, particularly PRS type (isolated or not), neonatal anatomic characteristics (cleft width, retrognathia degree), neonatal functional impairment severity (respiratory, orodigestive) and CP surgery procedure (one- or two-stage).

Our secondary objectives were to describe phonatory and morphological outcomes in a large monocentric cohort of PRS patients as they pass through the important period of adolescence and analyse early determinants of any phonatory or morphological sequelae that they may still have.

Finally, we also compared two surgical protocols for cleft repair (in one- or two-stage primary surgery) to assess the incidence of complications (palatal fistulae) and functional results in terms of facial morphology and phonation. Indeed, in our series, two protocols for CP repair were used. The first consisted of uranostaphylorrhaphy as per the Veau-Wardill technique [17] performed in a single intervention at age 9 months regardless of cleft width (Necker Hospital) and the second, intravelar veloplasty as per Sommerlad [18] performed at 6–8 months with closure of the hard palate during the initial surgery for narrow clefts or during a second surgery at 12–18 months (median age 15 months), for wider clefts (Trousseau Hospital). These two techniques and the results they confer have never been compared in patients with PRS, and their use in other types of cleft lip/palate remains controversial [19–24].

## Patients and methods

### Patients

All adolescents born between 01 July 1997 and 01 July 2007 (i.e., age 12–18 years during the study period) and admitted during the neonatal period to Necker Hospital or Trousseau Hospital for PRS were included. At that time, in France, the CP was part of the diagnosis. The PRS cases could be isolated or associated with underlying Stickler syndrome or with other minor bone malformations not threatening cognitive development. All children had been seen by the genetics team. Children with > 2 years of schooling delay were excluded, as were those who had serious undercurrent organic disease potentially deleterious to QoL.

The Île-de-France Ethics Committee II and the French national agency for medicines and health products safety (ANSM) approved the study protocol on 05 July 2016.

### Methods

Patients were examined during 1 day by the team of the French Rare Diseases Reference Centre. The examinations included the following:

(1) A phonation assessment performed by a speech therapist blinded to clinical data. This assessment included (a) a Glatzel mirror test for nasal air emission, responsible for hypernasality; (b) a vocal quality evaluation (hypernasality, vocal strength, hoarseness) and a phonation evaluation as per the Borel-Maisonny classification [25, 26]:

- Ph1: normal phonation
- Ph1/2: occasional nasal air emission (NAE); good intelligibility
- Ph2B: constant but non-audible NAE; good intelligibility
- Ph2/1: constant and audible NAE; improvement on effort
- Ph2: constant and audible NAE; no improvement on effort
- Ph2M: constant NAE hampering intelligibility or with synkinesis and/or forcing
- Ph2/3 or 3/2 (depending on the predominant mode): constant NAE; occasional compensatory mechanisms (glottal stops, pharyngeal friction); poor intelligibility
- Ph3: constant compensatory mechanisms; no intelligibility

The phonation assessment enabled the classification of phonatory impairment in three groups as follows: mild: no NAE on Glatzel mirror test, normal voice, Borel-Maisonny phonation 1/2; moderate: NAE on Glatzel mirror test, abnormal voice, Borel-Maisonny phonation 2b; severe: NAE on Glatzel mirror test, abnormal voice, Borel-Maisonny phonation 2m, 2/3 or 3.

(2) A morphological assessment performed by an orthodontist to analyse any chin projection or dentofacial defects and to note malocclusion according to Angle’s classification. This clinical assessment permitted classification of orthodontic abnormalities in three groups: minor/absent, moderate or severe. Thereafter, the moderate and severe orthodontic abnormality groups were pooled for further analyses because only one patient was in the latter group.

Furthermore, neutral portrait and profile photographs were taken the day of the examination, for independent, morphological analyses by two female assessors (a paediatrician and a maxillofacial surgeon) with blinding to all neonatal and surgical data. The assessors subjectively classified aesthetic results as good, moderate or bad with the possibility of a shared consensus judgment in cases of disagreement. Maxillomandibular lateral and frontal teleradiographs taken were analysed in another work. All the patients and their parents have signed an authorization to use these photos for clinic, teaching or research including scientific publications.

(3) An assessment of generic and specific (vocal and oral) QoL. Responses were collected during psychologist-led semi-directive interviews. Life traumas that may have modified QoL for the patient (death of a close family member, parental separation, etc.) were also identified.

The four following questionnaires were used:

*Child Oral Health Impact Profile (COHIP): oral QoL* The COHIP is designed specifically to assess the impact of orofacial abnormalities on QoL. It was first validated in the United States and Canada and thereafter in numerous countries. The full COHIP comprises 34 items, but a short-form 19-item version (COHIP-SF19) was developed by the instrument's authors for individuals aged 7–19 years [27]. The short and long versions show good equivalence. The COHIP-SF19 score is expressed as a global score and as oral, functional, socioemotional sub-scores. The initial scoring was reversed (never [0], to almost all of the time [4]) as it is done currently by the authors of the COHIP-SF19 [28]. Thus, the higher the score, the lower the oral QoL. The results from our participants were compared to those of other studies that used the COHIP-SF19, with score conversion when necessary. Of these studies, five reported global results for children in the general population, with similar age to our children, and were thus considered controls [27, 29–32]. We calculated the weighted mean of the COHIP-SF19 global score for the 1883 children from these five cohorts. No studies have used the COHIP in children with PRS. We compared our results to those of two series of children with craniofacial conditions from the Agnew et al. [33] series, we extracted the COHIP results for the 63 children aged 11–14 years and the 50 aged 15–18 years. These children had various types of orofacial cleft. We calculated the weighted mean of these two groups added to the 839 children from the Broder et al. series [27], who had various craniofacial malformations, without more precision in the publication, but mainly orofacial cleft because these children came from six expert cleft treatment sites in the United States.

*Voice Handicap Index (VHI): vocal QoL* The VHI is a QoL questionnaire exploring the physical, functional or emotional impact of voice disorders regardless of aetiology. The instrument was first validated in English in the United States and thereafter in a range of languages. The original VHI developed by Jacobson et al. included 30 items. In 2009, a shorter, international, nine-item version, called the VHI-9 international (VHI-9i), was derived for individuals aged ≥ 12 years. The VHI and VHI-9i have shown very good correlation. Responses to the nine questions are rated on a scale from 0 (never) to 4 (always) and the total score ranges from 0 to 36 [34]. Scores from 0 to 5, 6 to 10, 11 to 16 and 17 to 36 indicate no, mild, moderate and severe vocal disorders, respectively [35, 36]. These thresholds were used to interpret the VHI-9i results of the present cohort and compare them to results reported in four other series of adult patients with voice disorders [35, 37–39]. Of note, the pathological settings of these other studies were functional or organic dysphonia, settings different from the present cases of velopharyngeal insufficiency.

*KIDSCREEN-52: generic QoL* The adolescent version of the KIDSCREEN-52 was conceived for teens aged 12–18 years who may be healthy, chronically ill or socioeconomically disadvantaged. The KIDSCREEN questionnaires have been translated into 38 languages. Numerous European data are available for them, particularly control group results that supplement general population results obtained during the standardization of the instrument [40]. KIDSCREEN-52 is the complete version, comprising 52 items. Responses are chosen from a 5-point scale ranging from “never/not at all” to “always/extremely”. The KIDSCREEN-52 provides no global score. Its results are expressed for each of the 10 dimensions: “physical well-being,” “psychological well-being,” “moods and emotions,” “self-perception,” “autonomy,” “parent relations and home life,” “financial resources,” “social support and peers,” “school environment,” and “social acceptance (bullying).” The results from the adolescents in the present series were compared first to the T-score mean of 50 established in the general population used for the KIDSCREEN validation and second to the weighted mean for controls from different studies in which the KIDSCREEN-52 was used to evaluate children with disease. These latter values were reported in a meta-analysis [41]. From the cited studies, we retained only those for which the complete instrument was used and the participants were aged ≥ 12 years [42–48].

*Multiscore Depression Inventory for Children (MDI-C)* The MDI-C is a 15- to 20-min self-reporting instrument for measuring depression and its features in children aged 8–17 years. Its French adaptation was used here. The MDI-C has high test–retest reliability, good internal consistency and satisfactory concurrent validity with the Revised Children's Manifest Anxiety Scale [49]. It includes 79 short, true/false items worded so as to be easily understood by children. Its total score ranges from 0 to 79 and measures the severity of depression symptoms across eight subscales: “anxiety,” “self-esteem,” “sad mood,” “instrumental helplessness,” “social introversion,” “low energy,” “pessimism” and “defiance”. The instrument's raw scores are standardized, with distinct profiles for sex and age groups (8–10, 11–13, 14–17 years). For each of the subscales and the global score, there are three levels of symptomatology: global scores from 56 to 65, 66 to 75 and > 75 indicate mild, moderate and severe depression, respectively.

### Collected clinical data

- Socioeconomic status as per the French National Institute of Statistics and Economic Studies and geographic origin of the parent(s).
- Gestational age and anthropometric measurement at birth. Apgar score.
- Intrauterine growth restriction was noted when birth weight was below the 10^th^ percentile.
- Neonatal functional impairment severity classified as per Couly and modified by Cole [50]. Neonates for whom airway obstruction was treated with prone positioning and sucking difficulties with facilitation means (soft-nipple bottles, vertical positioning, thickened milk, etc.) were classified as grade 1; those for whom the airway obstruction was judged tolerable (partial pressure of CO_2_ < 50 mm Hg, oxygen saturation [SaO2] > 90% > 95% of the time, apnea hypopnea index < 10/h if polysomnography was done) in prone position and without ventilatory support but tube feeding was justified for > 8 days were classified as grade 2; and those for whom airway obstruction required intervention (intubation, nasopharyngeal airway, non-invasive ventilation or tracheostomy) were classified as grade 3. In Paris and between 01 July 1997 and 01 July 2007 (when the study participants were neonates), tracheostomy was the preferred technique for severe cases.
- Cleft palate width, classified as complete (or large), incomplete (or narrow) or soft-palate.
- Degree of neonatal retrognathia measured clinically as the distance between the two alveolar ridges in the awake, calm baby, cradled in a semi-seated position (45°; holding the back and neck) by the examiner. Measurements were classified as minor (inferior alveolar ridge < 5 mm from superior alveolar ridge), moderate (5–9 mm) or major (≥ 10 mm).
- Palate surgery: one- or two-stage primary surgery.
- Fistula sequelae.
- Secondary pharyngoplasty for rhinolalia resistant to speech therapy.
- Final diagnosis: isolated PRS; PRS within a collagenopathy; PRS associated with other malformations without cognitive disability.
- Severe congenital myopia > 10 diopters and/or retinal detachment.
- Hearing deficit treated with hearing aids.

### Statistical analysis

All calculations involved using SAS for Windows (v 9.4; SAS Institute Inc.). Statistical significance was set at *p* < 0.05. No adjustments for multiplicity were done. Descriptive analyses were conducted depending on the nature of the considered criterion. For quantitative data, this included the number of observed (and missing, if any) values, mean (standard deviation [SD]), median (interquartile range [Q1–Q3]) and range. For categorical data, this included the number of observed (and missing, if any) values and the number (percentage) of patients per class. Generalized linear models were used to identify prognostic factors and/or current explicative factors. Univariate comparisons between groups involved chi-square test, Fisher exact test or Mann–Whitney test depending on the nature of the studied criterion. One-sample *t*-tests and/or signed-rank tests were used for comparison with literature data. Finally, agreement between the assessment of patient photographs and the severity of the facial morphological abnormalities as determined under orthodontic examination was evaluated by the weighted kappa.

## Results

### General patient characteristics

A total of 101 patients were assessed; the 27 patients excluded were from 20 families lost to follow-up and 7 who did not wish to participate (3 for distance issues and 4 for inability or non-desire to participate). The remaining 74 patient families agreed to enrol their adolescents in the study. Of these, one patient was unable to undergo the complete speech evaluation and another was retroactively re-qualified as a case of isolated CP. Thus, the present study included 72 cases.

The 72 cases (38 females) were from 12 to 18 years old (mean [SD] age 14.4 [1.8] years), median 14). There were 59 (82%) cases of isolated PRS, 9 of Stickler syndrome and 4 of moderate bone malformation not related to a collagen gene defect (2 well-tolerated, molecularly non-explored epiphyseal dysplasias, 1 ossicle abnormality and 1 abnormality affecting the nasal bones). These 13 latter cases were labelled as “non-isolated PRS” for the purposes of this study. Eight of the nine Stickler syndrome cases had severe myopia (> 10 diopters) and two had deafness treated with hearing aids. Dominant inheritance was present in 10% and 38% of isolated and non-isolated PRS cases, respectively.

The socioeconomic distribution of the families reflected the general Paris region population, which has a larger proportion (42%) of managerial/executive and intellectual professions than the general French population (26.5%, corrected for working-age status of the families of the adolescents in the study). Similarly, the proportion of patients with at least one parent of foreign origin (33%) was similar to that of the general population of parents of children aged 11–18 years in the Île-de-France administrative region [51].

### Neonatal phenotype

Globally, the patients were born full-term (mean [SD] weeks of amenorrhea = 39.1 [1.4]) and at normal weight (mean birth weight = 3303 [532] g). The rates of prematurity (4.2%) and intrauterine growth restriction (11%) were similar to those of the general population [52–54]. Mean (SD) Apgar scores were 9.1 (2.1) at 1 min and 9.7 (1.0) at 5 min. At birth, 40% of the babies were transferred to neonatal intensive care or a specialized paediatric unit. By definition, all included patients were born with at least posterior CP (i.e., 61 [85%] with a large or complete CP, 6 [8%] with a narrow or incomplete CP and 5 [7%] with soft CP). Concerning retrognathia, 22 (31%) cases were classified as major, 31 (43%) moderate and 19 (26%) minor.

Functional impairment severity during the first weeks of life was grade 1 for 26 (36%) cases, grade 2 for 33 (46%) and grade 3 for 13 (18%). Tracheostomy was required for 9 of the 13 grade-3 cases (12.5% of the series). In total, 17 (25%) infants required enteral nutrition via a nasogastric tube or gastrostomy for ≥ 6 months, another 24 (35%) needed enteral nutrition via a nasogastric tube for < 6 months, and 27 (40%) were able to bottle feed (data missing for 4 cases). None were able to be breastfed.

Clinically, with the exception of a higher functional impairment severity grade with non-isolated than isolated PRS (*p* = 0.016), we found no statistically significant differences between the above early infancy data, in terms of isolated or non-isolated PRS (Table 1), sex, or considered age groups (12–14 and 15–18 years; data not shown).

**Table 1:** Neonatal characteristics of all adolescents and those with isolated and non-isolated Pierre Robin sequence (PRS)

	Total n = 72	Isolated PRS n = 59	Non-isolated PRS n = 13	*p* value*
Term (weeks), mean (SD)	39.1 (1.4)	39.1 (1.4)	39.5 (1.7)	
Birth weight (g), mean (SD)	3303 (532)	3248 (495)	3550 (637)	
Birth length (cm), mean (SD)	49.4 (2.4)	49.4 (2.1)	49.7 (3.6)	
Head circumference (cm), mean (SD)	34.6 (1.6)	34.4 (1.4)	35.7 (2.3)	
Cleft palate width				0.6757
Velar cleft	5	4	1	
Narrow or incomplete	6	6		
Large or complete	61	49	12	
Neonatal retrognathia severity				0.6800
Minor	19	17	2	
Mild	31	25	6	
Major	22	17	5	
Functional severity grade				**0.0164**
1	26	22	4	
2	33	30	3	
3	13	7	6	
Tracheostomy	9	6	3	0.3487
Secondary pharyngoplasty	18	16	2	
Fistula repair	13	11	2	

Bold indicates statistically significant results

*Chi-square or Fisher exact test

### Surgical itinerary

A one-stage surgery for CP repair was used in 57 (79%) cases. Specifically, 40 infants underwent uranostaphylorrhaphy at Necker Hospital and 17 intravelar veloplasty with closure of the hard palate in one-stage surgery at Trousseau Hospital. The remaining 15 (21%) infants underwent intravelar veloplasty with closure of the hard palate in two interventions at the latter hospital. In each hospital, nearly all surgeries were performed by a single surgeon. Follow-up surgery was required in 13 (18%) cases for fistula repair. PRS type, cleft width, neonatal functional impairment, severity grade, and surgery type were not risk factors for residual fistula. However, a “centre” effect was detected, with a significantly higher fistula rate among infants who underwent surgery at Trousseau Hospital versus Necker Hospital (odds ratio [OR] 12.9, 95% confidence interval [CI] [2.7, 83.7]). The rate of residual fistulas was clearly higher with intravelar veloplasty with closure of the hard palate than uranostaphylorrhaphy.

For 18 (25%) participants, secondary pharyngoplasty was required between age 6 and 12 years to correct persistent significant rhinolalia. PRS type, cleft width, neonatal functional impairment, severity grade, and surgery type were not risk factors for secondary pharyngoplasty (*p* > 0.05 for all).

### Current phonation

Speech assessments were completed for 70 of the 72 adolescents. Phonatory impairment was absent in 23 (33%), mild in 19 (27%), moderate in 28 (40%) and severe in none. Nasal air emission or hypernasality, as determined by a Glatzel mirror test and Borel-Maisonny classification, was the most frequently encountered sequela (present in 61 cases). The quality of phonation was identical for the adolescents with and without secondary pharyngoplasty. However, the former tended to have lower-quality vocal projection and inferior vocal strength than the latter. Phonatory assessment results are summarized in Table 2.

**Table 2:** Phonation assessment of all adolescents and those with isolated and non-isolated PRS and with and without secondary pharyngoplasty

	Total n = 70 (%)	Isolated PRS n = 57 (%)	Non-isolated PRS n = 13 (%)	No secondary pharyngoplasty n = 53 (%)	With secondary pharyngoplasty n = 17 (%)
Nasal air emission (Glatzer mirror)	43 (61.4)	39 (68.4)	4 (30.8)	32 (60.4)	11 (64.7)
Vocal quality					
Reduced vocal power	11 (15.7)	11 (19.3)	0	5 (9.4)	6 (35.3)
Vocal hoarseness	6 (8.6)	3 (5.3)	3 (23.1)	5 (9.4)	1 (5.9)
Vocal projection defect	10 (14.3)	9 (15.8)	1 (7.7)	5 (9.4)	5 (29.4)
Phonation quality					
Ph1	25 (35.7)	17 (29.8)	8 (61.5)	19 (35.8)	6 (35.3)
Ph1/2	17 (24.3)	16 (28.1)	1 (7.7)	12 (22.6)	5 (29.4)
Ph2B	5 (7.1)	4 (7.0)	1 (7.7)	4 (7.5)	1 (5.9)
Ph2/1	15 (21.4)	14 (24.6)	1 (7.7)	12 (22.6)	3 (17.6)
Ph2	8 (11.4)	6 (10.5)	2 (15.4)	6 (11.3)	2 (11.8)
PH2M; Ph2/3; Ph3/2; Ph3	0	0	0	0	0
Phonation outcome					
Normal phonation	23 (32.9)	16 (28.1)	7 (53.8)	17 (32.1)	6 (35.3)
Mild trouble	19 (27.1)	17 (29.8)	2 (15.4)	14 (26.4)	5 (29.4)
Moderate trouble	28 (40.0)	24 (42.1)	4 (30.8)	22 (41.5)	6 (35.3)
Severe trouble	0	0	0	0	0

NAE, nasal air emission

Ph1: normal phonation

Ph1/2: occasional nasal air emission (NAE); good intelligibility

Ph2B: constant but non-audible NAE; good intelligibility

Ph2/1: constant and audible NAE; improvement on effort

Ph2: constant and audible NAE; no improvement on effort

Ph2M: constant NAE hampering intelligibility or with synkinesis and/or forcing

Ph2/3 or 3/2 (depending on the predominant mode): constant NAE; occasional compensatory mechanisms (glottal stops, pharyngeal friction); poor intelligibility

Ph3: constant compensatory mechanisms; no intelligibility

On multivariate logistic regression considering PRS type, cleft size, neonatal degree of retrognathia, neonatal functional impairment severity, surgery type, centre and socioeconomic status (which may affect the quality of speech therapy management), only non-isolated PRS was associated with good phonation at adolescence. Given the counterintuitive nature of that finding, two different analyses were performed, both providing the same result. In the first, the 18 adolescents with a history of pharyngoplasty were included in the severe phonatory impairment group (OR 4.4, 95% CI [1.3, 16.9]), and in the second, the presence or absence of secondary pharyngoplasty was adjusted for (OR 4.0, 95% CI [1.1, 17.0]). These results suggest that two-thirds of children with PRS may retain low or moderate phonation difficulties but do not show determinants for them.

### Current morphology

**Fig. 1:**
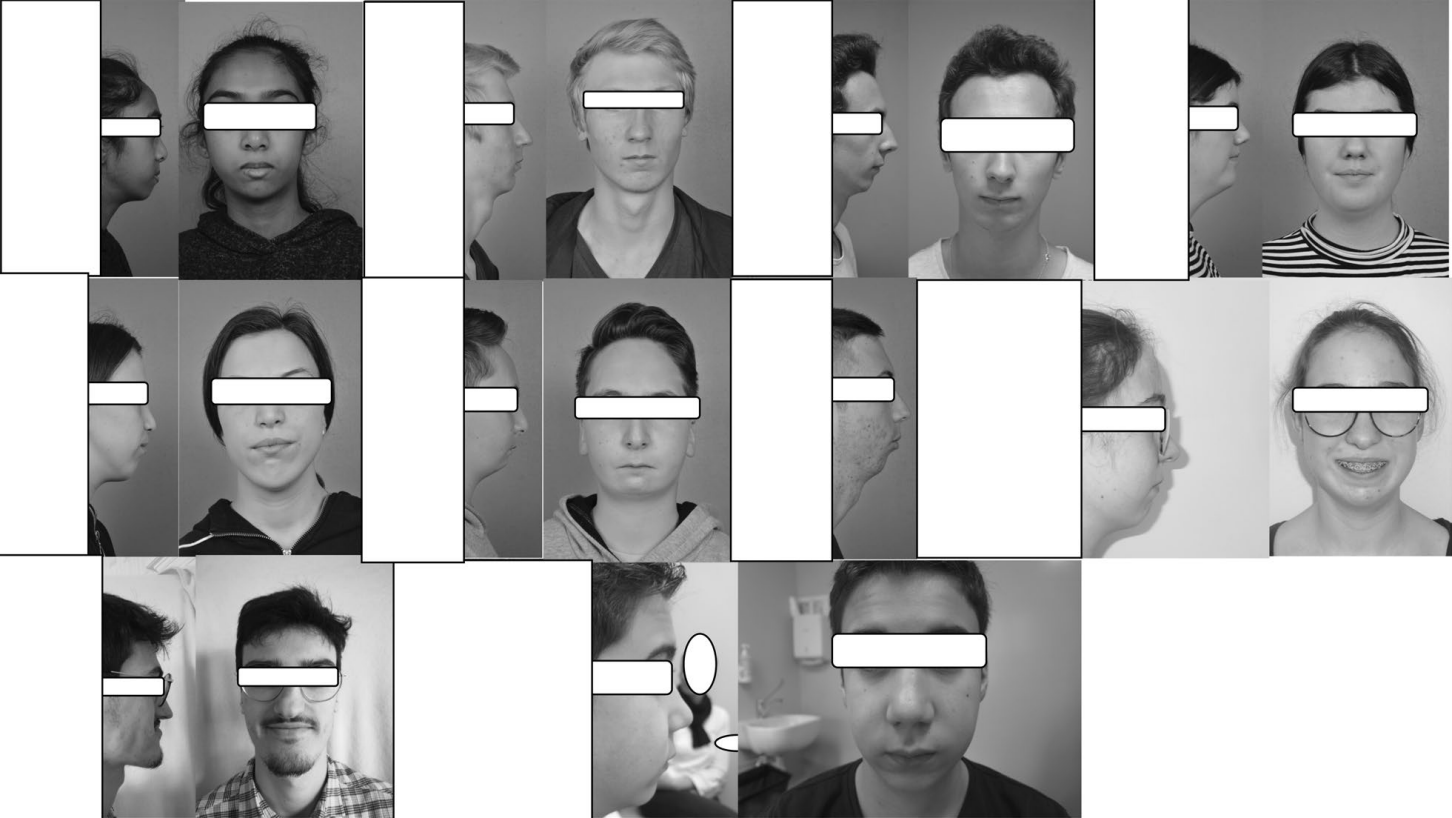
Photos of the 10 patients whose morphological results were considered to be the least harmonious

The portrait and profile photographs were interpretable for all but one adolescent. As subjectively judged by two of the team's physicians, 35 (49%) of the adolescents had harmonious faces (good aesthetic result), 26 (37%) mildly disharmonious faces with a somewhat receded chin (moderate aesthetic result), and 10 (14%) sufficiently disharmonious faces (poor aesthetic result: significantly receded chin, cervico-chin angulation defect, etc.) to justify a suggestion for genioplasty. The portrait and profile photographs of the 10 adolescents (7 males) judged disharmonious are presented in Fig. 1. Only one female had undergone condyloplasty before the study. She was subjectively (without knowledge of the previous surgery) placed in the moderate results group.

Of the 67 adolescents who had a complete orthodontic assessment, 35 (52%) were judged as having a good orthodontic state and 32 (48%) a moderate orthodontic state. None had a bad state (or severe abnormalities). More precisely, 45% of the adolescents had dentofacial disharmony, 31% an insufficiently projected mandible, 45% a narrow mandible and 39% a narrow maxilla. The orthodontic assessment in the present study was clinical; teleradiography results were the subject of another work, in prep. The results of the subjective aesthetic assessment and those of the orthodontic assessment were not in total agreement (weighted Kappa = − 0.006, 95% CI [− 0.226, 0.213]).

On multivariate logistic regression analysis considering sex, PRS type (isolated or not), cleft size (large or not), degree of neonatal retrognathia (minor, moderate or major), neonatal functional impairment severity (grade 1, 2 or 3) and surgery type (one- or two-stage), only the latter was identified as an early prognostic factor (*p* = 0.038) of aesthetic results at adolescence. Thus, correcting for other factors, the two-stage protocol (intravelar veloplasty with postponed closure of the hard palate when CP was large) was 4.7 times more likely to give good morphological results at adolescence than the one-stage intervention (uranostaphylorrhaphy or intravelar veloplasty with concomitant hard palate closure) (OR-associated 4.7, 95% CI [1.3, 20.7]). Of note, the aesthetic results of cases with Stickler syndrome did not differ from those of the other cases. The better aesthetic results conferred by the two-stage protocol did not carry over to the orthodontic results. Indeed, none of the considered factors (PRS type, cleft size, neonatal degree of retrognathia, neonatal functional impairment severity, surgery type) showed statistically significant prognostic value for that endpoint.

Neither neonatal retrognathia degree nor obstruction severity degree predicted less-satisfactory aesthetic results in the present series. Only the two-stage surgical protocol, the objective of which is to avoid maxillary growth defects, seemed to have a role in improving aesthetic results in adolescence.

### Oral-specific QoL (COHIP-SF19)

The mean (SD) global score for the COHIP-SF19 in our series was 17.5 (8.9). It was better with isolated than non-isolated PRS (mean 16.1 [7.3] vs 24.2 [12.5]). The global COHIP-SF19 scores for adolescents with isolated PRS were comparable to those of control cases and significantly better than those for children with craniofacial malformations in the other publications. The global COHIP-SF19 scores for adolescents with non-isolated PRS was significantly worse than that for control cases and close to that for children with craniofacial malformations in the other publications (Table 3). The sub-scores of the COHIP dimensions “oral health” and “social-emotional well-being” for the isolated PRS cases were similar to those for controls, but the sub-scores for the dimension “functional well-being” were worse than those for controls.

**Table 3:** Results of the Child Oral Health Impact Profile-short form for all adolescents and those with the isolated and non-isolated PRS, controls and other children with craniofacial conditions (weighted means of comparable series and controls)

	Total n = 72	Isolated PRS n = 59	Non-isolated PRS n = 13	Controls n = 1883 (5 series)	Craniofacial conditions n = 952 (2 series)
COHIP-SF 19 global score	17.5 (8.9)	16.1 (7.3)	24.2 (12.5)	15.4	25.7
Oral health	5.2 (3.1)	4.8 (2.6)	7 (4.2)	5.3	7.5
Functional well-being	3.2 (2.6)	2.8 (2.2)	4.7 (3.7)	1.9	4.3
Socio-emotional well-being	9.2 (5.8)	8.4 (5.2)	12.5 (7.2)	9.1	14.9

Data are mean (SD)

On multivariate analysis, we analysed the respective impact of several early factors (i.e., PRS type, CP width, grade of functional severity, and surgical type) and several current factors (i.e. morphological results, orthodontic results, and phonatory results). Multivariate analysis identified only PRS type as an early risk factor for poor oral QoL (*p* = 0.006). Specifically, the estimated adjusted mean was 7.9 points higher (worse status) with non-isolated than isolated PRS (non-isolated minus isolated OR 7.9, 95% CI [2.4, 13.3]). As expected, among the current determinants, good orthodontic results were correlated with better oral QoL. Thus, all other considered adjustment factors being equal, oral QoL was significantly lower in patients with orthodontically assessed moderate to severe facial abnormalities than only minor or no abnormalities (difference of 5.1 points [moderate to severe minus minor or no abnormalities], 95% CI [0.7, 9.5]).

### VHI-9i

For our 72 adolescents, the mean (SD) VHI-9i was 7.5 (5.4; median 6.5) (i.e., “mild vocal disorder” as per the scale established by the instrument's authors). The vocal QoL was better with isolated than non-isolated PRS in all three VHI-9i dimensions, even if the type of PRS was not a significant factor on multivariate analysis (Table 4). Of note, the evaluation by the speech therapist had pointed to a higher incidence of phonatory impairment in isolated than non-isolated PRS cases, which therefore contradicts the results of the VHI-9i. This finding may suggest that adolescents with non-isolated PRS perceive their own vocal disorders as being worse than they objectively are. The VHI-9i did not differ by sex age groups. The VHI-9i findings from the present series indicate a better vocal QoL than those reported in the literature for patients with voice-affecting pathologies (Table 5) [45, 46, 48, 56]. Of note, and logically, the adolescents assessed by the speech therapist as having no phonation impairment had a mean (SD) total VHI-9i score < 6 (5.8 [4.7]), which indicates a normal voice, whereas those assessed with low or moderate impairment had scores above that threshold (mean 7.9 [5.1]), which indicates a mild phonation disorder.

**Table 4:** Voice Handicap Index-9i for all adolescents and those with isolated or non-isolated PRS

	Total n = 72	Isolated PRS n = 59	Non-isolated PRS n = 13
Global score	7.5 (5.4)	6.8 (5.1)	10.8 (5.7)
Functional subscale	3.8 (2.7)	3.4 (2.6)	5.5 (2.7)
Physical subscale	2.8 (2.7)	2.5 (2.7)	4.0 (2.7)
Emotional subscale	0.9 (1.7)	0.8 (1.6)	1.2 (2.2)

Data are mean (SD)

**Table 5:** Voice Handicap Index -9i for adolescents with other troubles

	Functional dysphonia n = 26	Any voice pathologies n = 100	Vocal fold nodules PVU n = 24	Vocal fold nodules NPVU n = 13	Vocal fold polyps n = 61
Global score, mean (SD)	15.6 (6.7)	13.93 (7.8)	16 (7)	17 (8)	15 (8)

*PVU* professional voice user, *NPVU* non-professional voice user

No considered early factors (i.e., cleft width, functional severity, PRS type, surgery type, secondary pharyngoplasty status) affected long-term vocal QoL. As expected, vocal QoL was better for adolescents without than with phonatory difficulties (median 5.0 [Q1–Q3 2.0–9.0], n = 23, vs 7.0 [Q1–Q3 4.0–12.0], n = 47) but not to the point of significance for the global score (*p* = 0.098). However, the “functional” dimension of the VHI-9i differed significantly for these two groups (median 2.0 [Q1–Q3 1.0–4.0] and 4.0 [Q1–Q3 2.0–5.0]).

### Generic QoL

The results of the KIDSCREEN questionnaire suggested that overall, the adolescents had good generic QoL. The KIDSCREEN results can only be considered dimension by dimension. For both parametric and non-parametric analyses, none of the scores significantly differed from the instrument reference score of 50, except for the “moods and emotions” and “autonomy” dimensions, which were higher and lower, respectively. For the eight other dimensions, most of the scores were < 50, without significance. The comparison of the results from our series to those of the control group extracted from the meta-analysis by Silva et al. [28] showed a less favourable tendency, with significant differences for the dimensions “physical well-being,” “autonomy,” “financial resources,” “social support and peers,” and “school environment.” However, when only isolated PRS cases were considered, this statistical inferiority concerned only the dimensions “physical well-being” and “financial resources”. The results were less favourable for adolescents with non-isolated PRS, whose scores for the instrument's 10 dimensions ranged from 1 to 6 points less than those of adolescents with isolated PRS (Table 6).

**Table 6:** T-scores for the 10 dimensions of the KIDSCREEN questionnaire for adolescents with isolated and non-isolated PRS

KIDSCREEN dimensions	Isolated PRS n = 59	Non-isolated PRS n = 13	Total n = 72	Controls n = 23845	P1	P2
Physical well being					0.148/0.056 **0.0003/0.0001**	0.795/0.377 **0.016/0.008**
Mean (SD)	49.74 (7.73)	43.56 (7.45)	48.62 (7.99)	52.2		
Median (range)	49.63 (34.65–73.20)	42.53 (34.65–59.36)	47.08 (34.65–73.20)			
Psychological well being					0.790/0.419 0.589/0.419	0.747/0.794 0.940/0.794
Mean (SD)	50.38 (8.94)	46.73 (8.51)	49.72 (8.92)	50.3		
Median (range)	49.34 (32.80–68.49)	43.25 35.50–61.55)	47.12 (32.80–68.49)			
Moods and emotions					**0.001/0.002 0.001/0.002**	**0.0009/0.0007** **0.0009/0.0007**
Mean (SD)	54.40 (9.71)	50.46 (6.89)	53.69 (9.35)	50		
Median (range)	54.02 (31.42–70.91)	51.34 (38.86–62.06)	54.02 (31.42–70.91)			
Self perception					0.821/0.435 0.616/0.916	0.296/0.988 0.198/0.402
Mean (SD)	51.19 (8.67)	45.88 (7.07)	50.23 (8.61)	49.7		
Median (range)	49.76 (34.89–69.78)	44.58 (31.24–55.38)	49.76 (31.24–69.78)			
Autonomy					**0.031/0.013 0.023/0.005**	0.278/0.115 0.193/0.057
Mean (SD)	48.63 (9.63)	42.90 (9.25)	47.59 (9.76)	50.3		
Median (range)	46.85 (29.16–68.75)	40.54 (23.05–60.52)	46.01 (23.05–68.75)			
Parents relation and home life					0.241/0.271 0.078/0.078	0.728/0.731 0.346/0.301
Mean (SD)	49.55 (9.97)	(12.25)	48.56 (10.53)	50.8		
Median (range)	49.50 (30.18–65.87)	41.10 (23.19–65.87)	46.61 (23.19–65.87)			
Financial resources					0.339/0.636 **0.009/0.02**	0.446/0.676 **0.021/0.047**
Mean (SD)	49.01 (9.89)	48.08 (11.86)	48.84 (10.19)	52.1		
Median (range)	49.28 (23.24–62.86)	49.28 (23.24–62.86)	49.28 (23.24–62.86)			
Social support and peers					0.142/0.056 0.016**/0.006**	0.579/0.28 0.151/0.081
Mean (SD)	49.19 (11.19)	43.07 (8.43)	48.08 (10.95)	51.3		
Median (range)	46.66 (27.22–71.46)	42.20 (29.19–58.14)	45.08 (27.22–71.46)			
Social acceptance					0.347/0.129 0.057/**0.011**	0.676/0.41 0.184 /0.076
Mean (SD)	49.53 (8.56)	46.66 (10.09)	49.01 (8.85)	51		
Median (range)	48.61 (32.25–73.80)	45.34 (32.25–73.80)	48.61 (32.25–73.80)			
School environment					0.5035/0.181 0.698/0.826	0.128 /0.055 0.605/0.508
Mean (SD)	(7.59)	(9.23)	50.64 (8.07)	51		
Median (range)	48.07 (31.08–58.85)	48.07 (27.15–58.85)	48.07 (27.15–58.85)			

Bold indicates statistically significant results

P1: comparison of all patient scores and those of 1st line, to value 50 (one-sample *t*-test/signed-rank test) and 2nd line, to the weighted values of the controls extracted from Silva’s meta-analysis, 2019 (one-sample *t*-test/signed-rank test)

P2: comparison of the patients with isolated PRS scores and those of 1st line, to value 50 (one-sample *t*-test/signed-rank test) and 2nd line, to the weighted values of the controls extracted from Silva’s meta-analysis, 2019 (one-sample *t*-test/signed-rank test)

With multivariate analysis, we analysed the respective impact of early factors (i.e., PRS type and functional severity) and current factors (i.e., morphological result, orthodontic result, phonatory result, age, sex, socioeconomic status, existence of traumatic life events, COHIP and VHI scores). For the “physical well-being” dimension, non-isolated PRS but not initial function gravity was an early risk factor for reduced QoL. Thus, all other things being equal, that KIDSCREEN dimension was significantly worse with non-isolated than isolated PRS, with a difference of 6.3 points between the two groups (95% CI [− 11.4, − 1.3]).

Among the current determinants, only the results of the COHIP-SF19 score determined QoL for the “physical well-being” dimension. In other words, all other adjustment factors being equal, as oral QoL improved, so did generic QoL in the “physical well-being” dimension.

For the “self-perception” dimension, neither PRS type nor early functional severity were identified as early risk factors. Poor COHIP score, female sex and age 15–18 years were significant negative current determinants. In other words, all other things being equal, as oral QoL improved, so did generic QoL in the “self-perception” dimension. Self-perception was better with age 12–14 years than 15–18 years, with a difference of -5.9 points (15–18 minus 12–14 years, 95% CI [− 9.7, − 2.2]) and better for boys than girls, with a difference of > 5 points (males minus females, 95% CI [1.5, 9.0]).

These calculations were done for six KIDSCREEN dimensions (Table 7). Two dimensions, “physical well-being” and “parent relations and home life”, were affected by PRS type. All six of the dimensions were affected by the COHIP score, which tests some of the same aspects. Female and older adolescents may be more sensitive to differences and thus more susceptible to altered self-perception. To summarise, in the present study, generic QoL in cognitively unimpaired adolescents with PRS was mainly affected by the presence or absence of an associated malformation and by the oral comfort of the adolescents as assessed by the COHIP questionnaire.

**Table 7:** Significant neonatal risk factors and current determinants from the multivariate regression analysis of the KIDSCREEN questionnaire in the whole series

KIDSCREEN dimensions	Neonatal risk factors	Current determinants
Physical well-being	Non-isolated PRS	Oral quality of life
Psychological well-being	None	Oral quality of life
Parents relation and home life	Non-isolated PRS	Oral quality of life
Self-perception	None	Oral quality of life; female; 14–18 years
Autonomy	None	Oral quality of life
Social support and peers	None	Oral quality of life

### Depression

The mean responses to the MDI-C showed that severe depression was not present in the total, isolated PRS or non-isolated PRS adolescents. As a reminder, mild moderate and severe depression are indicated by MDI-C scores of 56–65, 66–75, and > 75, respectively. The mean (SD) total score was 47.6 (10) for all participants. Mean sub-group scores were 46.9 (9.7) with isolated PRS; 52.2 (11.3) with non-isolated PRS, 47 (8.5) for girls, 48 (11.8) for boys, 50.7 (11.5) with age 12–14 years, and 46 (8.5) with age 15–18 years. At the individual level, mild depression was present in 11 adolescents (scores 56–62) and moderate depression in 3 (scores of 66, 67 and 68). There were no cases of severe depression. Overall, 19% of the adolescents had symptoms of depression, but 0% had severe depression. These findings compare favourably to the general population, with rates for severe depression ranging from 4 to 5% and those for depressive mood or symptoms of depression reached 30%, especially during adolescence [55, 56]. Considering the instrument subscales in decreasing order of number of participants reaching the threshold score of 56, “anxiety” was the most frequent symptom (26.7%) followed by “self-esteem” (25.7%) and “instrumental helplessness” (25.3%). “Pessimism” and “defiance” were the least-frequent symptoms.

Multivariate regression analysis considering all factors that may affect depression symptoms (i.e., morphologic, orthodontic, and phonatory difficulties, age, sex, socioeconomic status, traumatic life events, poor oral or vocal QoL), only the COHIP score was a significant determinant (*p* < 0.0001) and socioeconomic status was a weakly significant determinant (*p* 0.05). In other words, as oral QoL and socioeconomic status decrease, depression scores increase.

## Discussion

We present here an analysis of the current situation for 91% of the now-adolescents who, at birth, were treated for PRS in two tertiary hospitals (merged today) in Paris, France. Our study benefits from a high participation rate of adolescents, who were neonates at a time when follow-up was shorter and less strict than it is today.

Overall, our results show that adolescents who were born with PRS but without cognitive impairment had a normal QoL, on average. We confirm that children with non-visible malformations have less difficulties regarding self-esteem and mood than those with visible craniofacial malformations or scars [14]. Nevertheless, in more detail, the comparison of our series to a pooled series of control patients of the same age showed worse generic QoL for our adolescents than control patients in dimensions regarding physical health and relations to others. Having non-isolated PRS was the main factor reducing generic QoL for our adolescents. Dimensions evaluating self-confidence (“self-perception,” “autonomy”) and social relations (“parent relations and home life,” “social support and peers”) were affected in adolescents for whom the medical issue remained present (non-isolated PRS). The “self-perception” and “social support and peers” dimensions reflected best what adolescents expressed in interviews with psychologists (i.e., that they have been and/or are targets of mockery from their fellow students). Also “anxiety” and “self-esteem” were the most frequently affected depression aspects of the MDI-C dimensions.

Contrary to our initial hypothesis, neither facial aesthetic nor phonatory sequelae directly affected generic QoL in our adolescents. These findings may reflect the perception of these sequelae as minor by the patients although professionals noted them. It may illustrate a good capacity for resilience, surely the result of good medical outcomes and pertinent familial and medical support. This capacity for resilience may also be reflected in the KIDSCREEN “moods and emotions” dimension, in which our adolescents scored higher than the norm.

However, more attention is needed for PRS cases with associated malformations (Stickler or others). This remains true for patients with no cognitive disability or grade retention/schooling delay because potential suffering in this population may go undetected. We feel that psychological support through childhood and adolescence is important for these patients.

Our results agree with those reported by Basart et al. in 2017 [16], the only other team to have evaluated generic QoL in adolescents with PRS. However, that study involved only 17 adolescents who completed the Pediatric Quality of Life Inventory over the Internet. Because of the lack of a psychologist to guide and deepen responses, the responses they gathered may not reflect the full breadth of their patients' perceptions. Parental presence may also have affected the responses gathered, thus reducing their value. The authors also reported a tendency toward lower values in adolescents with non-isolated than isolated PRS.

Oral QoL remains an important issue in adolescents with PRS. Indeed, the generic QoL of our adolescents was well-correlated with their oral QoL, which underlines the impact of dental and orthodontic problems on QoL and self-confidence in this setting. As in generic QoL, non-isolated PRS was a risk factor for poor oral QoL, even though the objective, professional evaluations of facial morphology, orthodontic state and phonation were not worse with non-isolated than isolated PRS. Here too, it appears that the simple awareness of having a chronic disease may have a larger impact as compared with certain objective elements describing current PRS sequelae. The oral QoL results we report here cannot be compared to other series of adolescents with PRS. They can be compared to those of an Australian study [33] of children with various types of clefting because the authors used the same tool than we did. Their children had poorer oral QoL than ours, but the proportion of children with PRS in the Australian study is not known. As we do, the previous authors underlined the link between oral and generic QoL. This shared observation calls for vigilance as to the management of oral issues in PRS patients.

Although two-thirds of our patients retained mild to moderate phonation troubles, their vocal QoL was within the norm, and was higher than those of adults with voice pathologies. Of note, in our series, children with significant phonatory sequelae had already undergone surgery. The only determinant for vocal QoL was the intensity of current phonation issues. Our results seem better than but nonetheless are in the same direction as those of studies that specifically investigated effects of velopharyngeal insufficiency (VPI) on QoL with the new VPI Effects on Life Outcomes (VELO) instrument (estimation from the child and the parent[s] themselves) [57, 58]. The VELO is not yet translated or validated in French. Correlations between VELO results and objective speech assessment by a speech therapist remain controversial [59].

Residual phonation disorders of children with PRS and CP is a major issue, with incidences ranging from 13 to 47% across series (interviewer's observations, ages at time of the assessment, specialized examinations by a speech therapist, assessment techniques, voice-correction surgery rate, etc.). Our results confirm this high frequency. Unfortunately, we identified neither early (e.g., PRS type, cleft size, surgery type) nor current (particularly socioeconomic status of the family) risk factors for these phonation disorders. Importantly, the hyperlaxity present in certain patients with Stickler syndrome did not seem to affect velopharyngeal insufficiency. We could not evaluate the quality of any speech therapy our participants may have had. Of note, results were similar for adolescents with and without secondary velopharyngeal surgery. We did not collect perioperative data on palatal muscles, the characteristics of which may play a predictive role in later phonation disorders. However, our colleagues from Trousseau hospital did so and found no predictors for phonological outcomes [26]. The difficulties we encountered for predicting velopharyngeal insufficiency or phonation disorders are shared with other authors. Those who compared phonatory sequelae in children with PRS to those with isolated CP showed a higher rate of disorders in the former but were unable to identify determinants [23, 26, 60–64]. Recently, Logjes et al. [65] showed that the only determinant for speech sequalae in Robin sequence, as in isolated CP, was the width of the cleft. Differences across these results may be due to variable ways of measuring the type and size of the CP. The standardization of evaluation methods would be of great interest for comparing results among studies.

Facial growth may be assessed in several ways, including simple clinical observation by an experienced specialist, orthodontic examination or X-rays and angle measurements. In PRS, this aspect is essential for two reasons. The first is a question of pathophysiology. A neonatal retrognathia with an origin in bone anatomy should persist more than one with a functional origin secondary to a defect in foetal oral mobility. Thus, the course of mandible growth in these children is a retroactive diagnostic element. The second is a question of therapy. If the bone anomaly is the sequence starting point, early mandibular distraction is more justified than if not. In our assessments, 13.5% of adolescents were considered candidates for esthetical surgery, particularly for boys, because the chin plays a role in the perception of virility. However, we found no correlations between the morphological aspects in adolescence and neonatal retrognathia degree, respiratory impairment severity, or PRS type. Thus, from our findings, we are not able to identify infants who will need genioplasty at adolescence. These results suggest no anatomical justification for early mandibular distraction, and, additionally, genioplasty at adolescence is both a simple surgery and free of side effects on dentition.

The only predictor of better facial morphological results at adolescence was the two-stage surgical protocol. However, intravelar veloplasty with closure of the hard palate resulted in a higher rate of residual fistulas as compared with uranostaphylorrhaphy. In the literature, the frequency of residual fistulas after palatal surgery varies from several percentage points to 36% [26, 60, 62, 63, 66]. These secondary surgeries are sufficiently frequent to merit early parental information.

## Conclusions

Morphological or phonatory impairments remain non-rare in adolescents with PRS, whether isolated or associated with bone or collagen disorders, but do not appear to be directly responsible for altered QoL. These adolescents show self-confidence and social-relations fragilities, especially those with non-isolated PRS, who find themselves circumscribed within the status of chronic disease. Building upon the past, when the neurological and cognitive sequelae resulting from neonatal airway obstruction were reduced, the medical community must now turn its attention to bettering long-term functional and psychological results for PRS patients by improving therapy protocols and follow-up, notably for those affecting the oral aspects of the disease.

## Data Availability

All the data are available at the clinical research department of Imagine Institute of Paris, and the Reference Centre for Rare Diseases “Séquence de Pierre Robin et troubles de succion-déglutition congénitaux” in Necker University Hospital, Paris.
